# A comparison of emergency department medical records to parental self-reporting of traumatic brain injury symptoms

**DOI:** 10.2217/cnc-2017-0017

**Published:** 2018-04-06

**Authors:** Audrey McKinlay, Alanah Lin, Martin Than

**Affiliations:** 1 Melbourne School of Psychological Sciences, University of Melbourne, Melbourne, Victoria 3010, Australia; 2 Department of Psychology, University of Canterbury, Christchurch, New Zealand; 3 School of Medicine, Christchurch Campus, University of Otago, Christchurch, New Zealand; 4 Department of Emergency Medicine, Christchurch Hospital, Christchurch, New Zealand

**Keywords:** concussion symptoms, medical records, traumatic brain injury

## Abstract

**Aim:**

Studies have shown Emergency Department (ED) recording of traumatic brain injury (TBI) cases to be poor.

**Methods:**

Parents of children aged 2–12 who attended an ED with injury to the head completed a concussion checklist which was compared with medical records.

**Results:**

ED medical records commonly used head injury (HI), concussion, minor-HI and mild-HI without distinction between TBI and superficial HI. Recalled symptoms included vomiting, blurred vision and headaches versus headaches, fatigue and feeling sick from parents who reported more concussive symptoms. More cases of TBI were identifiable from parental recall compared with medical records, which recorded fewer symptoms for diagnosis, prognosis and statistical reporting of TBI.

**Conclusion:**

Clear guidelines need to be implemented to improve retrospective diagnosis for incidence gathering and future clinical use.

Traumatic brain injury (TBI) is a frequently occurring event, with young children representing a particularly vulnerable group. Previous measures of TBI incidence in New Zealand suggest that around 856 per 100,000 person years [1], or even as high as 1100 per 100,000 person years, occur in the 5–14 age group [2]. In the USA, 475,000 incidents involving children 0–14 years of age occur annually [3]. These incidence rates highlight a pressing need for research into the treatment of TBI. However, this cannot be carried out without accurate identification of TBI.

Previous studies have suggested that there is a routine failure to diagnose or document TBI in hospitals [4,5], with up to 56% of mild TBI cases failing to be noted in the Emergency Department (ED) records [5]. In addition, retrospective recall is often used to elicit TBI prevalence in the community for subsequent validation against medical records. However, recent studies have highlighted poor recall accuracy [6], especially for early childhood injury [7], and even where loss of consciousness is used as an identifier, recall for early injury is poor and false recall is prominent [8]. Studies measuring incidence rates rely almost solely on medical records for validation [1–3], hence inadequate recording of TBI may contribute to any underestimation of the true incidence rates and provide an inaccurate source for validating self-reported TBI. In 2003, the Centers for Disease Control and Prevention (CDC) published recommendations for the identification of mild TBI [9], including in the infant and young child; however, there has been a lack of studies describing what terminology is used in an ED to describe a TBI event, and no research that compares the information in medical records to parental recall of TBI symptoms. As has previously been reported, younger children rely on a parent or caregiver to interpret their behaviors and to communicate symptoms for them, with less overt symptoms likely to be under reported [10]. From a health intervention perspective, accurate measures of the incidence rates are crucial to support any proposed change in policy or legislation.

The first aim of this study was to evaluate how ED hand written medical records communicate an incident of childhood mild TBI. A second aim was to compare the degree with which signs and symptoms that are recorded in ED medical records differ to those subsequently recalled by parents. We predict that ED staff will record fewer signs and symptoms compared with the subsequent recall by parents given a symptom checklist. Comparing medical records with parental recall will provide information about what ED clinicians may not record in the medical records and/or what they may not ask about in their examination, and also highlight the importance of medical records in cross-validating patient and parental report/recall.

## Methods

### Participants

The sample population was children between 2 and 12 years of age presenting to an urban ED at Christchurch Hospital, Christchurch, New Zealand, and who had experienced an injury to the head as noted in the ED daily attendance list. Children within this age range rely on a parent or caregiver to present them for treatment, with falls being the most common mode of injury for this age group [2]. Individuals were excluded if parents had sought medical attention for noninjury-related conditions. The study was conducted over an 18-month period with potential participants prospectively identified using the ED daily attendance list. Parents of these children were then contacted by phone by hospital staff within 1 week of ED attendance and invited to participate. If they agreed, parents were then sent information regarding the purpose of the study and initial data collection material. Parents gave consent for participation, and where appropriate, the child also assented to participate. Due to ethical requirements, information about children whose parents did not agree to participate was withheld by the hospital. Of those who agreed to participate, 37.3% provided a list of their child's concussion symptoms and were included in the study. The study had approval from the Upper South A Regional Ethics Committee.

In addition to superficial injury to the head, individuals were included in the study if a medical diagnosis was available that stated that a head injury (HI), concussion or mild TBI had occurred. Cases where a loss of consciousness had occurred and where one or more additional symptoms of concussion were listed, but where no TBI diagnosis was noted, were also included as TBI (n = 6 cases), providing 107 confirmed cases of mild TBI. Participants stated their ethnicity as New Zealander 79.5%, Maori 8.6%, Pacific Islander 3.3%, European 1.3%, Asian 2.7% and other ethnicities 4.6%; with age and sex of the children shown in Table 1.

**Table 1.** **Age and sex of participant children.**

**Age**	**2 Years**	**3 Years**	**4 Years**	**5 Years**	**6 Years**	**7 Years**	**8 Years**	**9 Years**	**10 Years**	**11 Years**	**12 Years**
Male	17	25	10	10	6	12	4	8	6	7	5

Female	15	4	5	3	5	6	3	7	9	5	4

### Procedure

Consenting families were asked to complete a concussive symptom checklist that was mailed to the parents within 1 week of injury and was collected by a research assistant the following week. Parents were asked to indicate whether or not their child had any of the symptoms on the checklist at the time of injury. The checklist items were uplifted from the Rivermead Concussive Checklist [11], with the following additional items: anxiety, easily distracted, loss of memory and loss of consciousness, used in a dichotomous format. Parents were also asked to indicate whether they had observed, or if their child reported, any additional symptoms not listed. With parental consent, a research assistant also reviewed the medical records for each child and recorded all notes made by the ED consulting doctor. A total of 176 participants were included in the analysis.

### Statistical analysis

Descriptive statistics (counts and percentages) were used to provide information regarding the demographic characteristics of the children and the concussive symptoms reported by each of the groups. To accommodate the small quantity recalled for some symptoms, the Fisher's exact test was used to analyze statistical significance of any difference found between medical records and parent recall. A difference was considered significant at p < 0.05.

## Results

### Medical record terminology

The words most commonly used in the medical records to denote that a TBI event had occurred were HI (43/176, 24.4%), concussion (37/176, 21.0%), minor HI (21/176, 11.9%) and mild HI (14/176, 8.0%). However, there were few if any distinctions between these terms in the notes. Glasgow Coma Scale (GCS) was recorded for 72 children, 60 were at 15, 8 were at 14 and 4 between 14 and 12. Loss of consciousness (LOC) was recorded for 34 children, with LOC and GCS being recorded for 15 children.

### Parent symptom recall

Parents were most likely to report headaches (n = 103), fatigue (n = 98) and feeling sick (n = 86), as shown in Table 2, and on average they reported more symptoms than doctors recorded. The top three symptoms most likely in medical records (and as percent of parent recall) were vomiting (87%), blurred vision (58%) and headaches (51%). Symptoms that were reported in both medical records and recalled by parents were slightly lower, with doctors recording 77% of vomiting, 39% of blurred vision and 39% of headaches that were recalled by parents.

**Table 2.** **Number of symptoms recalled by parents and recorded in medical records, and percentage of agreement between medical records and parental reporting of symptoms.**

**Symptom**	**Parent symptom recall or symptoms in medical records**			**Symptoms recalled and in medical record**	

	**Parent recall (n)**	**Medical records**		**Medical records**	

		**n**	**Percentage of recall**	**n**	**Percentage of recall**
Vomiting	53	46	86.8	41	77.4

Blurred vision	33	19	57.6	13	39.4^†^

Headaches	103	52	50.5^†^	40	38.8^†^

Loss of memory	48	19	39.6^†^	16	33.3^†^

Fatigue	98	47	48.0^†^	31	31.6^†^

Feeling sick	86	31	36.0^†^	27	31.4^†^

Confusion	61	18	29.5^†^	12	19.7^†^

Dizziness	72	13	18.1^†^	12	16.7^†^

Loss of consciousness	41	7	17.1^†^	5	12.2^†^

Restlessness	36	8	22.2^†^	4	11.1^†^

Other	9	24	266.7^†^	1	11.1^†^

Irritability	59	11	18.6^†^	6	10.2^†^

Anxiety	47	2	4.3^†^	2	4.3^†^

Difficulty paying attention	42	0	0.0^†^	0	0.0^†^

Easily distracted	33	0	0.0^†^	0	0.0^†^

Easily frustrated	41	0	0.0^†^	0	0.0^†^

Forgetful	23	0	0.0	0	0.0^†^

Sensitivity to light or noise	27	0	0.0	0	0.0^†^

Sleep problems	40	0	0.0	0	0.0^†^

^†^Difference of number of symptoms reported is statistically significant p < 0.05.

### Retrospective diagnosis using records & recall

Using the CDC Criteria for TBI [9] (see Supplementary Information 1) and symptoms recorded by doctors and recalled by parents, a total of 92 TBI cases were retrospectively identified out of the 176 injury to the head cases, or 147 if fatigue, vomiting or irritability were regarded as diagnostic of TBI in children. Often insufficient symptoms were present in the medical records to retrospectively make a TBI diagnosis. Only 32 retrospective TBI diagnoses could be made using medical records alone. In contrast, using parental recall of symptoms a total of 86 retrospective diagnoses could be made, as shown in Table 3.

**Table 3.** **Number of times symptoms recalled by a parent or in medical records fulfilled the Centers for Disease Control Criteria for traumatic brain injury.**

**Source of symptoms**	**Number of TBI events**	**When including F, V, I**
Medical records provide sufficient criteria	32	90

Parental recall provides sufficient criteria	86	137

Combined medical records and parent recall	92	147

F: Fatigue; I: Irritability; TBI: Traumatic brain injury; V: Vomiting.

### Consistency of retrospective diagnoses

Table 4 shows that of the 107 TBI cases identified in the ED, only 67 (62.6%) could be retrospectively diagnosed using symptoms from medical records. A total of 40 out of 84 events not fulfilling CDC Criteria were diagnosed as TBI in the ED (37.4% of TBI). A similar trend was shown using the alternative expanded symptom checklist criteria.

**Table 4.** **Emergency Department traumatic brain injury status compared with identification using Centers for Disease Control Criteria for traumatic brain injury from combined parental recall and medical records.**

**ED status of injury to the head cases**	**CDC Criteria for TBI**		**When including F, V, I**	

	**Fulfills criteria**	**Does not fulfill criteria**	**Fulfills criteria**	**Does not fulfill criteria**
Mild TBI (n = 107)	67	40	99	8

Superficial (n = 69)	25	44	48	21

CDC: Centers for Disease Control and Prevention; ED: Emergency Department; F: Fatigue; I: Irritability; TBI: Traumatic brain injury; V: Vomiting.

## Discussion

Parents reported more symptoms than were recorded in the medical records. Using parent checklist criteria, 86 out of 92 TBI events meeting CDC Criteria were identified, whereas the medical records only provided enough information to identify TBI in 32 events. The most commonly recorded symptoms by ED clinicians were vomiting, blurred vision and headaches. In contrast, parents were most likely to report fatigue, feeling sick and headaches. Commonly used terms by the clinicians to describe trauma to the head were HI, concussion, minor HI and mild HI, and all of these were applied to both TBI and superficial injury to the head events.

Obtaining information in an ED setting with major time constraints can be extremely difficult, especially from children, who often need a third party (such as a parent) to provide or interpret the information. However, the quantity of information that is typically noted in the medical records, which would justify the ED doctor's diagnosis or for another doctor to make the same diagnosis, was minimal at best. Further, there is insufficient information to provide a source of validation for self-reported TBI later in life. When compared with parents, only vomiting was recorded by a doctor in over 50% of the incidents where parents had recalled it. It is probably not feasible for the ED attendant to ask about every symptom that a child may have exhibited after a traumatic injury; however, priority should at least be given to symptoms that are helpful for either establishing or ruling out a diagnosis of TBI (as well as symptoms that may indicate palliative care) (loss of consciousness, amnesia, confusion, irritability, lethargy and vomiting) and prognosis (dizziness, headache, amnesia and GCS score) [12–14].

The number of symptoms noted in medical records were in contrast much lower than provided by parents, who were shown in this study and previous studies to have a high rate of reporting [15,16]. This is perhaps due to parents being present at the time of injury or soon after, and having access to more sources than the ED staff as to ascertain what happened. In addition, a parent is likely to spend more time with the child before and after the injury, hence have better insight into changes in the child's behavior and mental state, but which may not be clinically relevant in an ED setting.

Multiple reasons may also account for the reduced or inaccurate information in the hospital records. Two of the most likely are that this information was gathered by the doctor and thought clinically unimportant, hence not written down, or the doctor did not gather this information at all. In both of these circumstances, it is possible that many ED clinicians are unclear of the definition of TBI, and this may account for why many injuries diagnosed as concussion did not have any concussive symptoms recorded by either the doctor or the parent to support this. This finding is similar to a study by Mackenzie and McMillan where knowledge of postconcussive symptoms was examined among 30 general practitioners; the results showed that even among health professionals there was no clarity regarding the signs and symptoms of a TBI [17]. An alternative explanation could be that the doctors may have asked about the symptoms, but the parents failed to report these at the time of the ED examination.

Considering these possible explanations, it is of no surprise that this study was congruent with previous studies that have been conducted with adult populations and have reported that accuracy of diagnosis regarding traumatic brain injuries was lacking [4,5]. In this study there was considerable variance between doctors on the definition of TBI-related terminology. Variability of terminology may cause confusion for parents regarding the injury and expected outcomes. For example, previous research has demonstrated that there is a perception within the general public that concussion represents a more minor injury than when terms such as mild HI or mild TBI are used [18].

The misdiagnosis of TBI for patients can have serious implications as short-term effects of TBI include fatigue [19], memory problems, irritability, anxiety, dizziness, increased sensitivity to noise and light, as well as headache, with findings being common at 1 month postinjury and lasting up to as long as a year [20]. For children especially, the existence of postconcussion symptoms [15], behavioral problems [21,22] and decline in school-readiness skills have been documented following even mild TBI [23]. As such, after a TBI, there are multiple factors inhibiting a child from recovering and getting back to everyday activities. A lack of accurate diagnosis from a parental perspective may make recovery harder due to poor public awareness of the effects of TBI [17,24]. A concern for the cases of TBI that are not diagnosed is that parents may not be aware of indicators of deterioration requiring urgent medical attention, or that a future injury of this kind may require hospital treatment.

The major strength of this study is that we were able to collect information from a large unselected sample. Further, we were able to examine the ED medical records unbiased by the knowledge that these were to be reviewed. However, the study also had a number of limitations. We were unable to gather the information from the parents immediately following the injury event prior to being seen at the ED. In many cases, the parents may have been provided with an information sheet giving a list of concussive symptoms to be aware of which may have influenced their report. Parents may also have been reporting symptoms that had manifested after they had been seen by the ED doctor. Further, only 37.3% of those who agreed to participate to complete the symptom checklist perhaps introducing a bias toward parents whose children were continuing to exhibit concussion symptoms.

From this study, it is difficult to tell if it is the doctor not asking the right questions, or if the parents are not telling the doctors when asked, or if symptoms are just not being recorded. The implications of this is that there is a lack of evidence in hospital records to accurately diagnose TBI for epidemiological studies, and it may also have contributed to the low accuracy of diagnosis found in this study. It is clear that there is no consistent guideline for the usage of terminology regarding TBI or its diagnoses. Symptoms recorded in the ED setting need to be more consistent to aid in clinical and surveillance use. The CDC does provide forms specifically for recording TBI-related symptoms, so perhaps hospitals should develop similar forms to assist ED doctors recording in all injury to the head cases [25]. Since parents were consistently more likely to report concussive symptoms, a similar checklist to the one used in this study could be provided in injury to the head cases to parents on entering the ED to aid recall before they are seen by the doctor. Both methods would assist ED staff to diagnose TBI versus a superficial injury to the head, and aid in the efficient documentation of symptoms for future reference.

## Conclusion

Details from ED medical records are commonly used to inform health service provision, postinjury clinical services and insurance requirements, as well as validate patient report. As such, the detail presented in these medical records goes beyond the immediate need of an ED. Procedures need to be consistently implemented to enable ED staff to accurately and efficiently describe TBI-related diagnosis and patient presentation for future reference.

**Summary points**

- Studies have shown Emergency Department recording of traumatic brain injury cases to be poor.
- Emergency Department medical records commonly used head injury (HI), concussion, minor HI and mild HI without distinction between traumatic brain injury and superficial HI.
- Clear guidelines need to be implemented to improve retrospective diagnosis for incidence gathering and future clinical use.
